# Editorial: Acute pancreatitis infection: Epidemiology, prevention, clinical characteristics, treatment, and prediction

**DOI:** 10.3389/fcimb.2023.1175195

**Published:** 2023-03-21

**Authors:** Wandong Hong, Jingye Pan, Hemant Goyal, Maddalena Zippi

**Affiliations:** ^1^ Department of Gastroenterology and Hepatology, The First Affiliated Hospital of Wenzhou Medical University, Wenzhou, Zhejiang, China; ^2^ Intensive Care Unit, The First Affiliated Hospital of Wenzhou Medical University, Wenzhou, Zhejiang, China; ^3^ Department of Surgery, University of Texas Health Sciences Center, Houston, TX, United States; ^4^ Unit of Gastroenterology and Digestive Endoscopy, Sandro Pertini Hospital, Rome, Italy

**Keywords:** acute pancreatitis, infection, severe acute pancreatitis, infected pancreatic necrosis, prediction, pathophysiology

Acute pancreatitis (AP) is a common gastrointestinal disease and has been increasing in recent years worldwide (Hong et al., 2020). This special issue Research Topic highlights advances in the epidemiology, prevention, treatment, and prediction of the severity of AP.

## Prevalence and etiology

Gallstones, hypertriglyceridemia, and alcohol are the most common causes of AP (Lee and Papachristou, 2019). However, the etiology composition varies across the globe. He et al. enrolled 5146 adult AP patients from 2011 to 2017 to investigate variations in the disease etiology. They found an upward trend in the diagnosis of alcohol-related AP while a decrease in the incidence of acute biliary pancreatitis (ABP). Moreover,the composition ratios of ABP and hypertriglyceridemic-AP were affected by seasons and festivals, which could be due to an increase in fatty food consumption.

Gallstones accounts for the most common etiology of AP in China, which can progress to severe sepsis or shock if not treated in timely fashion. However, not all patients with gallstones develop the symptomatic disease during their lifetime. The available data is scarce about the development of ABP in patients with symptomatic gallstone diseases such as cholecystitis and choledocholithiasis. Guo et al. conducted a retrospective case-control study and found that age, diabetes, gallbladder wall thickness, gallstone diameter, coexisting choledocholithiasis, direct bilirubin and white blood cell count are significantly associated with an increased risk of concomitant ABP in patients with symptomatic gallstones. They developed a nomogram consisting of these indices with good discrimination predicting the ABP occurrence.

Chronic pancreatitis (CP) is a fibroinflammatory disorder with irreversible scarring to pancreatic parenchyma (Kothari et al., 2023). Its’ common symptoms include abdominal pain, nausea, weight loss, steatorrhea, and diabetes (Kothari et al., 2023). It is well known that both alcohol and tobacco are the common risk factors for CP development. Hao et al. compared the clinical characteristics of smoking and alcohol-related chronic pancreatitis. They found that the development of diabetes and pseudocyst was significantly more common and earlier in smokers than in alcoholic patients. In addition, Steatorrhea was found to be significantly more common in smokers. The findings of this study indicate that smoking-related CP develop early and has higher risk of developing complications than idiopathic CP. The smoking related-CP should be considered as a new independent subtype of CP an these patients should be managed aggressively to prevent the development of complications.

## Pathophysiology

Aseptic inflammation is the initial manifestation of injury in AP. However, the pathogenesis of AP has not been fully understood although trypsin-centered theory of AP has been proposed for more than a century. Many additional studies have been conducted to understand the pathogenesis and mechanism of AP development. Besides pathological calcium signaling and endoplasmic reticulum stress, the role of damage-associated molecular patterns (DAMPs) and neutrophil extracellular traps (NETs) was found to play a significant role in activating, signaling and recruiting inflammatory cells and the adaptive immune response giving rise to the sterile inflammation. In order to offer a better understanding of the pathophysiological mechanism and new insights for future investigational AP treatment options, the review by Zhou et al. describes the role of DAMPs and NETs, their interplay in the pathological progress of AP and potential targeted therapeutic modalities against DAMPs and NETs. They suggested that DAMPs could encompass the activation and recruitment of innate immune cells, mediate the formation of NLR Family Pyrin Domain Containing 3 (NLRP3) inflammasome, participating in forming NETs by activated neutrophils. Targeted therapeutic modalities against DAMPs and NETs, such as blocking DAMPs signaling, decreased expression of extracellular DAMPs, increased expression of intracellular DAMPs, and blockage of NETs formation may be helpful for severe AP.

AP is an inflammatory disease often accompanied by the occurrence of systemic inflammatory response syndrome (SIRS) and compensatory anti-inflammatory response syndrome (CARS). However, it is a topic of debate whether SIRS and CARS occur in succession or in parallel when AP arises. Through bioinformatics analysis, the study by Liu et al. on experimental model found that SIRS and CARS occur in parallel. They also found that toll-like receptor 2 (TLR2) could mediate the dysregulation of inflammatory response in AP and can be a novel therapeutic target to manage these patients.

## Prediction of disease severity

While most patients with AP have a milder course and the disease is self-limiting, but about 20% of the AP patients progress to severe disease (Hong et al., 2020). Patients with severe acute pancreatitis (SAP) often need to be transferred to the intensive care unit after developingorgan failure. Therefore, it is essential to recognize predictors for severe disease in the early phase of AP, to select those patients who would benefit most from early interventions (Hong et al., 2019; Hong et al., 2020). The study by Li et al. compared the performance of interleukin-6 (IL-6) and C-reactive protein (CRP) as a potential predictor of SAP, organ failure, pancreatic necrosis, infected pancreatic necrosis, and mortality. Their study revealed that IL-6 is a better predictor of mortality and infected pancreatic necrosis in AP (AUC 0.75 vs. 0.70 and 0.81 vs. 0.65, respectively). Multiple past studies have reported that repeating serum amylase levels has no value in assessing the clinical progression and prognosis of AP. However, the study by Hong et al. found a a non-linear association between the amylase day 2/amylase day 1 ratio and incidence of SAP by logistic regression with restricted cubic spline analysis. Integration of Day 2/Day 1 amylase ratio ≥0.3 as 1 point to the bedside index for severity in acute pancreatitis (BISAP) score (BISAP-A score) significantly improved its diagnostic utility compared to the original BISAP score (AUC, 0.86 versus 0.83).

Artificial intelligence is increasing being used in the clinical setting for disease prediction or aiding in making decisions Hong et al. However, implementation of such data remains challenging because of the low interpretability of machine learning results. The study by Hong et al., proposed an interpretable random forest model with a sensitivity of 93.8%, specificity of 82.8%, and a diagnostic accuracy of 83.9%. The local interpretable model-agnostic explanations (LIME) plot was used to explain the individualized prediction. Simultaneously, a logistic regression model with a nomogram consisted of albumin, serum creatinine, glucose, and pleural effusion was also developed as a comparison. The study by Yin et al. developed an automated machine learning model based on the gradient boost machine algorithm to predict the AP severity achieving a sensitivity of 0.583 though its’ specificity and accuracy were >0.95.

## Management

It is well-known that early enteral nutrition (within 24-72h of admission) in AP patients is associated with decreased rates of complications and mortality (Arvanitakis et al., 2020). Jin et al. performed a retrospective study on 98 patients with predicted severe acute gallstone pancreatitis who received early therapeutic endoscopic retrograde cholangiopancreatography (ERCP). They found that starting early enteral nutrition (within 48 h) was associated with a decrease in in-hospital mortality, length of stay, need for intensive care, and hospital care costs.

Post-inflammatory pancreaticopleural fistula (PPF) is a rare but serious complication of acute and chronic pancreatitis. The primary pathophysiology is disruption of the main pancreatic duct (MPD) or smaller pancreatic ducts, leading to leakage of pancreatic juice into the pleural cavity. In a prospective study by Jagielski et al. on 22 PPF patients found that ERCP with MPD stent placement for passive transpapillary drainage is effective with procedure success rate of 95.45%. The one-year success rate of endoscopic therapy was achieved in 86.36% patients.The results of this study shows that PPF can be treated effectively with preferential MPD drainage.

The data about antibiotic prophylaxis forendoscopic drainage of post-inflammatory pancreatic and peripancreatic fluid collections is scarce. Jagielski et al. performed a randomized, double-blinded, and placebo-controlled trial to investigate the role of periprocedural antibiotic prophylaxis in endoscopic transmural drainage in 62 patients with symptomatic pancreatic and peripancreatic fluid collections. Their results revealed that prophylacticantibiotics are not needed for pancreatic and peripancreatic fluid collections if the endoscopic transmural drainage was achieved successfully.

Infected pancreatic necrosis (IPN) is one of the primary determinant of severity in AP due to the high mortality of up to 32% (Petrov et al., 2010). Concomitant multi-drug resistance Gram negative bacteremia (MDR-GNB) can occur in AP with IPN in clinical practice. Only a few studies have examined the impact of MDR-GNB bacteremia in IPN patients. In a case–control Wu et al. found that IPN patients with MDR-GNB bacteremia was associated with a higher mortality rate than that without MDR-GNB bacteremia (OR 8.976, 95% CI 1.805 –44.620, *p*=0.007).

The importance of fungal infection in patients with IPN and pseudocysts is increasing being recognized. Patients with SAP and intra-abdominal fungal infection suffer higher in-hospital mortality than patients with intra-abdominal bacterial infection alone (Trikudanathan et al., 2011). Protracted broad-spectrum antibiotic therapy and a prolonged ICU stay are associated with an increased fungal infection risk (De Waele et al., 2019). However,the clinical presentation of fungal infection in acute necrotizing pancreatitis is somewhat variable and nonspecific (Trikudanathan et al., 2011). The review by Otsuka et al. summarizes recent advances in the clinical influence and molecular mechanisms of pancreatic fungal infection on the development of SAP as well as the efficacy of anti-fungal therapy. Their review summarizes that nucleotide-binding oligomerization domain 1 (NOD1) and toll-like receptor 4 (TLR4) sense the bacteria leading to a robust production of pro-inflammatory cytokines causing intestinal barrier dysfunction and translocation of gut bacteria into the pancreas. In the other hand, recognition of β-d-glucans by Dectin-1 play a important role in translocation of gut fungi into the pancreas due to leakiness of the barrier because Candida cell walls are strong stimulators for Dectin-1 expressed in macrophages and dendritic cells (Netea et al., 2015).

Further large scale *in-vivo* and *in-vitro* studies are needed to elucidate the underlying mechanism of AP and its local complications such as infected pancreatic necrosis. Furthermore, studies with high-quality evidence are needed to develop AP prognostication models using simple laboratory markers and clinical scoring systems. High-quality multicenter randomized controlled trials are required to determine whether prophylactic antibiotics have a role in a specific group of SAP, necrotizing pancreatitis, or endoscopic drainage of post-inflammatory pancreatic and peripancreatic fluid collections (Crockett et al., 2018). More studies are also needed to determine the optimal management of IPN (Working Group, 2013).

## Author contributions

WH wrote the first article draft and JP, HG, MZ helped to revise the article. All authors contributed to the article and agreed with the last version to be accountable for the content of the work.
